# The provision of TB and HIV/AIDS treatment support by lay health workers in South Africa: a time-and-motion study

**DOI:** 10.1186/1478-4491-12-18

**Published:** 2014-04-04

**Authors:** Willem A Odendaal, Simon Lewin

**Affiliations:** 1 Health Systems Research Unit (HSRU), Medical Research Council of South Africa (MRC) and Department of Psychology, University of Stellenbosch, P O Box 19070, Tygerberg 7505, Cape Town, South Africa; 2 Health Systems Research Unit (HSRU), Medical Research Council of South Africa (MRC) and Global Health Unit, Norwegian Knowledge Centre for the Health Services, St Olavs plass, PO Box 7004, N-0130 Oslo, Norway

**Keywords:** Antiretroviral therapy, Community health workers, HIV/AIDS, Lay health workers, Low-income, Primary health care, TB, Time-and-motion study, Treatment support

## Abstract

**Background:**

Lay or community health workers (LHWs) are an important human resource in primary health care, and contribute to improving access to care. However, optimal use of LHWs within the health system is often hampered by a poor understanding of how this cadre organizes its work. This study aimed to better understand how LHWs organize and structure their time in providing treatment and adherence support to people on TB treatment and/or antiretroviral therapy (ART) in South Africa.

**Methods:**

Fourteen LHWs participated across three low-income peri-urban communities in Cape Town. Each LHW was observed by a researcher for one day, and data collected on each activity and the time spent on it. Data were summarized in the following categories: travel to the patient’s home, waiting time and patient contact time.

**Results:**

Ninety-seven attempted visits to patients were observed, and patients were located in 69 of these. On average, LHWs conducted six visits per day, each lasting an average of nine minutes. Forty-six percent of the observed time was spent with patients, with the balance spent on ‘non-contact’ activities, including walking to and waiting for patients. The average walking time between patients was 8 minutes (range: 3 to 15 minutes). Activities during visits comprised medical care (that is ensuring that medication was being taken correctly and that patients were not experiencing side-effects) and social support. Other tasks included conducting home assessments to determine risks to treatment adherence, and tracing patients who had defaulted from treatment.

**Conclusions:**

Because of their tasks and working environment, LHWs providing support to people on TB treatment and ART in South Africa spend a substantial proportion of their time on ‘non-contact’ activities. Programme managers need to take this into account when developing job descriptions and determining patient case-loads for this cadre. More research is also needed to explore whether these findings apply to other tasks and settings. Strategies should be explored to mitigate the challenges that LHWs experience in locating and supporting patients, including the use of new technologies, such as mobile phones.

## Background

The optimal use of human resources to provide quality and equitable health care is an on-going concern globally [1]. This concern is highlighted in recent recommendations by the World Health Organization (WHO) on optimizing health worker roles for maternal and neonatal health [2]. These recommendations consider the extent to which less highly trained health workers can be enabled to safely and effectively provide services which are otherwise performed by more highly trained cadres.

In many settings, community members serving as lay or community health workers (LHWs) - health workers who perform diverse functions related to health care delivery but who have no formal professional training [3] - are an indispensable part of primary health care services across a range of programmes [4-6]. LHWs are seen as a way of improving access to health services and mitigating health workforce deficits in resource-constrained settings [7]. This cadre plays an important and growing role in providing treatment and adherence support for people living with HIV/AIDS and tuberculosis (TB) [8], and in improving maternal and child health [5]. A recent systematic review has examined the global evidence from randomized trials on the effects of LHWs in primary and community health care for maternal and child health, and the treatment of infectious diseases. This review showed that the use of this cadre probably leads to an increase in the number of people with TB who are cured but probably makes little or no difference in the number of people who complete preventive treatment (usually with isoniazid) for TB [5].

In South Africa, the current re-engineering of primary health care (PHC) services envisages that the approximately 60,000 LHWs in the country will become part of local ward-based PHC outreach teams led by a professional nurse. This re-engineering not only aims to optimize the efficiency and impact of LHWs, but also to expand their support for people on treatment. It is planned that treatment support, currently focused on people on TB treatment and antiretroviral therapy (ART), will be extended to other chronic conditions, such as hypertension and diabetes [9]. Given this expansion in the roles of LHWs, it is particularly important to better understand how this cadre’s work is organized and how they structure their time.

### Time-and-motion studies

Any task shifting, restructuring or development of new models to improve service delivery needs to include the careful management of human resources [1,10,11]. Ideally, context-specific evidence should guide performance expectations and staffing norms [12]. One tool for collecting such evidence is the ‘time-and-motion’ study (T&M), defined as the independent and continuous observation and recording of staff activities and the time spent on these [13,14]. In these studies, the independence of the observer counters the tendency in self-reporting to over-report activities that participants view as more desirable, for example in relation to their managers’ expectations [15]. Hadleya and Roques [16], for example, found major discrepancies between nurses’ self-reporting of daily activities, in which they merely cited their formal job descriptions, and the results of a T&M study of these nurses’ work.

T&M studies are labour-intensive and are therefore commonly done with small samples of participants: the observer will shadow a participant for a specified period of time and minutely record the time and activities observed [14]. Activities are usually grouped into categories that reflect the core components of participants’ work, and the aggregated time spent per category is then reported.

Although LHWs are used widely across a range of settings, few published T&M studies have focused on this cadre. The available literature reports mostly T&M studies with professional cadres, such as nurses and doctors, working in healthcare facilities [16-18]. Two T&M studies were found with non-professionals, but these workers were based in healthcare facilities and therefore do not provide information on the time spent on different activities by LHWs in community settings [19,20]. There are several possible reasons for this lack of research: firstly, LHWs are often not seen as part of the formal health system and there may therefore be less interest among health care managers in how they spend their time; and secondly, the challenging circumstances in low-income neighbourhoods may make it difficult to conduct T&M studies with LHWs who deliver healthcare in patients’ homes.

## Objective

This study aimed to better understand how LHWs organize and structure their time by recording the activities undertaken by them, and the time spent on these, in providing treatment and adherence support to people on TB treatment and/or antiretroviral therapy in South Africa.

## Methods

### Study setting

South Africa remains the country with the largest HIV/AIDS epidemic in the world, with approximately 5.6 million people living with HIV/AIDS in 2009 [21]. Globally, it is also ranked as having the third highest TB burden with an estimated new TB case incidence of 981 per 100,000 population [22]. TB-HIV coinfection is a major and growing concern in South Africa with an estimated 128,457 people coinfected in 2010, which is 14,000 more coinfections in 2010 than in 2009 [23].

The study was conducted in the Cape Metropole, one of the health sub-districts of the Western Cape Provincial Department of Health (WCDoH). The number of TB cases in the sub-district is one of the highest in South Africa, with 28,658 cases reported in 2011, or a new smear positive incidence of 752 per 100,000 population, compared to the national estimate of 500 per 100,000 [24]. In 2010, the HIV prevalence in the Cape Metropole was 19.1%, which is lower than the national prevalence of 30.2%.

To access LHWs, we approached a large nongovernmental organization (NGO) that is contracted by the WCDoH to employ LHWs to provide treatment and adherence support to people on treatment for TB and HIV. In the past, treatment and adherence support for TB and ART patients have been provided separately in South Africa, often by different NGOs, with the consequence that two LHWs may be assigned to patients who are coinfected. The LHW programme of the study NGO was one of the first to integrate TB and ART support, with one LHW assigned to support patients who are coinfected with both diseases. The LHWs were expected to work four and a half hours per day and paid a stipend of 120 USD per month - this stipend was set by the health authorities and transferred to the NGO for disbursement to the LHWs. It should be noted that LHWs did not receive any additional funds in relation to transport costs for patient visits.

The tasks undertaken by LHWs are summarized in Table 1. Apart from reporting to the NGO, the LHWs had weekly case management meetings with the staff from the publicly funded primary health care facility in their community. During these meetings, new patients were assigned to them and they also had the opportunity to give feedback to facility staff on issues arising during their visits to patients.

**Table 1:** Summary of key lay health worker (LHW) tasks and responsibilities

**LHW task**	**Description**
*Home assessments*	● After a patient is assigned to a LHW, the LHW completes a socio-economic assessment form with the patient. This form also records the patient’s enablers and possible barriers to treatment adherence.
*Treatment and adherence support visits*	● Observation of treatment taking is provided to TB patients, as part of the direct observation of treatment (DOT) strategy.
	● Visits also include medical (pill counts; checking for treatment side-effects; helping patients to manage their treatment dosages) and social (help with HIV non-disclosure and psycho-social problems) support to patients.
*Door-to-door health promotion visits*	● Health promotion and illness prevention visits are made to each home in the community.
	● Visits include the dissemination of general health information on TB and HIV and on TB screening. LHWs refer residents to the healthcare facility for TB/HIV testing and for other medical problems identified during visits.

Three low-income communities in the Cape Metropole were purposefully selected because these were sites for the integrated LHW-service offered by the NGO. The sites had similar demographic profiles (Table 2): residents were ethnically diverse and most lived in informal housing, with its characteristic alleys and complicated house numbering system. Site 3 differed in that it was a slightly wealthier community that also included formal housing areas.

**Table 2:** **Community demographic profile**[25-27]

**Site**	**Male**	**Unemployment rate**	**Income (< 320 USD per month)**
**1**	55%	33%	79%
**2**	48%	45%	74%
**3**	49%	27%	50%

### Participants

Permission was obtained from the NGO to recruit LHWs in each of the sites. Following the study briefing, 14 of the workers volunteered and consented to participate, representing 44% of the LHWs across the sites. All of the LHWs who participated were females and resided in the communities in which they worked; their demographic profile is described in Table 3.

**Table 3:** Demographic profile of lay health workers (LHWs) participating in the study

**Site**	**Average age (years)**	**Educational level**
		**(proportion with more than 11 years of schooling)**
**Clinic 1**	31	25%
**Clinic 2**	38	60%
**Clinic 3**	48	25%

No selection criteria for participation were used as it was understood that their duties were similar. LHWs were requested to obtain verbal consent from patients prior to the researcher’s visit with them.

### Data collection and analysis

Each worker was accompanied by one researcher for one day. Researcher 1, a female registered for a post-graduate degree in public health, collected data in Site 1, whilst researcher 2, a male with a post-graduate degree in research psychology, collected data in Sites 2 and 3. Each researcher piloted the data collection tool with one LHW, and the format for recording activities and time was then revised. The pilot data were excluded from the results below.

The researchers used a stop watch and recording sheet to record each activity and the time spent on it (for more detail see Table 4 below). A total of 33 hours of observations of LHWs were recorded. During these observations, the researchers talked with the LHWs but did not provide any feedback on the activities observed. The data for each LHW were summarized into three main categories: walking, waiting and patient-contact time, and aggregated per site. The data were captured and analysed in Microsoft Excel 2010. As door-to-door health promotion visits were undertaken routinely in only one of the study sites, these data are not presented.

**Table 4:** **A typical T&M day spent with a** lay health worker (**LHW**)

	
I (the researcher) arrived at 08:30 at the LHW’s house, and we promptly left for her visits. It was interesting that she covered the stationery and materials from her NGO in a magazine to hide the purpose of her visits from the scrutinizing eyes of neighbours. Her first patient was not at home, a pattern that was repeated three more times that day. As the LHW had to protect the confidentiality of the patient, she could not check with the neighbours on the patient’s whereabouts. She said that she would return later in the week, hoping to find the person at home then.	Each interaction with a patient started off with a conversation about general issues before the LHW asked about the patient’s health. For those who had started treatment recently, she asked specifically about side-effects. A pill count would follow and in one instance much time was spent on educating an illiterate patient to manage her pill box. Each visit concluded with encouraging words to the patient. I was deeply moved by the LHW’s compassion for a 12-year-old living with HIV, and her calming and reassuring interaction with the mother of the child.

Ethical approval (EC09-028) was obtained from the Medical Research Council, South Africa.

At the end of the study, the researchers presented the results to the NGO and the three LHW teams.

## Results

Across the three sites, the LHWs had an average monthly patient case-load of 42 patients each. Typically, the LHWs would conduct their visits from 08:00 to 12:30 and managed to visit between six and fourteen patients each day. Visits to patients who were adherent and responding well to the treatment were generally very short and some of the workers were therefore able to conduct a large number of visits. The LHWs themselves planned who they were to visit each day. They were also often requested by staff of the local health facility to trace people who had defaulted from treatment and to conduct home assessment visits (see Table 1). Assessment visits lasted an average of 19 minutes.

Across the sites, the frequency of visits to ART and coinfected patients ranged from weekly to once a month. In Sites 1 and 2, adherent TB patients, whether HIV positive and on ART or not, self-administered their TB treatment. In these two sites, the local health facility had developed a system for categorizing all patients based on their adherence to TB treatment and/or ART. Patients were categorized as ‘green’ if they were assessed as adherent to treatment and therefore needed less LHW support, or as ‘red’ if they were considered to be at risk of treatment non-adherence and/or had experienced severe side-effects and therefore required intensive LHW support. This system aimed to rationalize visit frequency in Sites 1 and 2: ‘red’ category patients were visited at least once a week while ‘green’ category patients were visited monthly. In Site 3, patients receiving DOT for TB were seen daily. In all three sites, TB, ART and coinfected patients who received their treatment in the community or self-administered their treatment, and who were assessed by either the facility staff or LHWs as non-adherent, were referred back for facility-based treatment.

Table 5 presents the T&M data for each site and the averages across the sites. The percentages reflected for ‘*Time with patients’* are a proportion of the total observed time aggregated per LHW and then per site.

**Table 5:** Summary of how lay health workers (LHWs) spent their time during treatment and adherence support visits in the community

**Site**	**Average walking time between patient visits**	**Average duration of home visit**	**Average number of visits done per day**	**Time with patients as % of the observed time**	**Total number of patients found at home/Total number of visits done (% of visits with patients found at home)**
**Site 1**	6 minutes	8 minutes	4	27%	18/28 (64)
**Site 2**	3 minutes	7 minutes	10	64%	38/51 (75)
**Site 3**	15 minutes	11 minutes	5	48%	13/18 (72)
**Average**	**8 minutes**	**9 minutes**	**6**	**46**%	**69/97 (71)**

Across the sites, the average proportion of LHWs’ time that was spent with patients was 46%; the remaining 54% was ‘non-contact’ time. There was some variation across the sites in the activities constituting ‘non-contact’ time: in Site 1, 73% of LHWs’ time was ‘non-contact’ time and this was spent on walking to (42%), and waiting for patients (31%). LHWs in Sites 2 and 3 respectively spent 36% and 52% of their time walking to patients; no waiting time was recorded in these sites. This can be explained by the fact that the LHWs had to obtain patients’ permission, prior to the ‘T&M day’, for the researcher to be present during the visit. This was strictly observed in Sites 2 and 3, and these patients were thus expecting the LHW. Locating patients and their homes was a common problem across the sites. For example, in one instance the local clinic requested the recall of eight patients. The assigned LHW spent 105 minutes looking for these patients and located only four of the addresses provided. Only two of these patients were found at home, and the LHW spent a total of 18 minutes with these patients, which constituted 17% of the observed working time on that day.

## Discussion

These results highlight the challenges that LHWs experience in extending the services, usually provided by health professionals at healthcare facilities, to the homes of patients [5,6]. The high proportion of LHWs’ time that was categorised as ‘non-contact time’ should not be seen as indicating poor productivity on the part of LHWs. Rather, these data reflect the difficulties of finding patients’ homes in settings with no formal street addresses as well as the likelihood of finding patients at home during working hours.

Our findings are similar to those reported by Mallidou *et al*. [19] and Qian *et al*. [20] in their studies of non-professional carers in health care facilities. In these two studies, only 52% and 31% of the observed time respectively were spent in direct contact-care with patients. Non-contact time, whether travelling to a patient’s home, waiting for patients or undertaking administrative duties, is an inevitable part of LHWs’ daily activities in most settings. In community settings, assigning patients to LHWs based on their home’s proximity to the LHW’s home may reduce walking time. However, there are cases where patients express a preference for a particular LHW who does not live close by. An example from Site 3 in this study illustrates this challenge: here the walking time to a ‘patient-preference home’ was 70 minutes, resulting in approximately one quarter of the LHW’s day being spent on one patient. These detailed data on how LHWs organize and structure their time could contribute to the development of wider explanations of how LHW programmes impact on health outcomes, including TB and ART outcomes, and the factors affecting the implementation of LHW programmes at scale [5,28]. The challenges encountered by LHWs may also contribute to poor retention rates for this cadre and may, in some settings, also impact on recruitment.

This study found that LHWs conduct an average of six visits per day, close to the 5.6 visits per day in urban settings that has been recommended by the National Department of Health, as part of the re-engineering of primary health care in South Africa [29]. However, a ‘once size fits all’ approach for case-loads across different neighbourhoods may not be useful as this does not take geographical and logistical differences into account. This is illustrated by the differences between the three sites (Table 5): the walking time in high density communities such as informal settlements (Sites 1 and 2) was substantially lower than that in formal, lower density urban areas (Site 3), where homes are further apart. Local estimates of travelling, waiting and visit times should be used to determine the ideal number of patients to assign to each LHW.

Our results emphasize the need for careful and considered human resources management for LHWs [2,28]. Appropriate job descriptions and performance assessment criteria are important tools for increasing the effectiveness of LHW programmes [30], but need to be based on the realities of LHW’s work. Our findings support Vale’s [6] view that evidence of LHWs’ experiences and working conditions is needed to counter unrealistic employment conditions and expectations from their employers. Data from T&M studies such as this one should prompt agencies that employ and support LHWs to consider strategies to optimize the delivery of LHW services. The following approaches could mitigate some of the challenges recorded in this study:

• Match LHWs and patients based on smaller geographical areas so as to reduce travel time for LHWs

• Establish systems to identify and prioritize ‘at-risk’ patients who may benefit from more intensive LHW support, so as to keep the case-load manageable for each LHW

• Explore the use of mobile phones to improve communication between LHWs and patients, including around the scheduling of support visits, so as to reduce the number of unnecessary home visits that LHWs make [31]

• Explore the use of mobile phone-based GIS systems to mark the location of patients’ home so that they can easily be found by the health services in the future [32]

This study has several limitations. The small scale of the research means that the findings should be generalized with caution to other settings and programmes. Also, the proportion of patients found at home in Sites 2 and 3 might be higher than that is seen typically, as the LHWs needed to obtain patients’ consent in advance for the researcher to visit. The true proportion of patients found at home in relation to visits done may therefore be lower than reported here. Finally, it is possible that the LHWs changed their behaviours and practices as a consequence of being observed. For example, LHWs may have wanted to demonstrate to the researchers their challenging working conditions and therefore chosen, on the observation day, to visit problematic patients who lived furthest from the clinic. However, all of the patients visited had been assigned to the LHWs for treatment support, and the data is therefore likely to reflect how LHWs spend their time on a day-to-day basis. Also, while independent observations might shape the behaviours of those observed, this approach counters the tendency of participants to overreport activities that they view as more desirable [15,16].

## Conclusions

This study begins to address the paucity of data from low- and middle-income countries on how LHWs in community settings organize their work. It highlights some of the challenges LHWs face in delivering care in low-income neighbourhoods, and the importance of taking context into account when planning LHWs’ work and developing (national and local) norms and performance assessment criteria for this cadre. Employing agencies need to understand the realities of LHWs’ working conditions before creating job descriptions, case-loads and performance criteria that may be unrealistic. Also, while it may not be possible to address the frustrations of not locating patients’ homes or not finding patients at home, these frustrations may be mitigated both through supportive supervision and through the use of new technologies, such as mobile phones [33,34].

There is a risk that focusing on the time that LHWs spend on different activities could be seen as a way of making LHW care more quantifiable and target-driven - an approach aptly described as the ‘corporate paradigm’ of care [6, p. 21]. In this study, the researchers were struck not by the obvious frustrations that LHWs experienced in locating patients (as reflected in non-contact time) but, rather, by the genuine care that was observed in all of the LHWs’ interactions with their patients. LHWs are, and will remain, an important way of mitigating health workforce deficits in resource-constrained settings [28], and careful management is needed to support them in their roles and to optimize their time with patients.

## Abbreviations

ART: Antiretroviral therapy; DOT: Directly observed therapy; GIS: Geographic information system; HIV/AIDS: human immunodeficiency virus/acquired immune-deficiency syndrome; LHW: Lay health worker; NGO: Nongovernmental organization; TB: Tuberculosis; T&M: Time-and-motion; WCDoH: Western Cape Department of Health; WHO: World Health Organization.

## Competing interests

The authors declare that they had no financial or non-financial competing interests in conducting and publishing the study.

## Authors' contributions

WO developed the data collection instrument, collected and analysed the data and drafted the manuscript. SL conceived the study, participated in its design and data analysis, and in drafting the manuscript. Both authors read and approved the final manuscript.

## Authors’ information

WO is a senior scientist at the Health Systems Research Unit of the Medical Research Council of South Africa. He holds a Masters’ degree in Research Psychology and has conducted previous formative evaluations on lay health worker services. SL holds a PhD in sociology as applied to health and is a senior specialist scientist at the Health Systems Research Unit at the Medical Research Council of South Africa. He is also a senior researcher at the Norwegian Knowledge Centre for the Health Services. SL has published widely on lay health workers, and is the first author of a Cochrane review on the effectiveness of lay health workers.
